# Age and sex differences in paediatric tuberculosis care for child contacts

**DOI:** 10.7189/jogh.16.04100

**Published:** 2026-03-20

**Authors:** Allison Boretsky, Farhana Amanullah, Hamidah Hussain, Amyn A Malik, Meredith B Brooks

**Affiliations:** 1 Department of Global Health, Boston University School of Public Health, Boston, Massachusetts, USA; 2 Boston Children’s Network Specialty Physicians, Boston, Massachusetts, USA; 3 Interactive Research and Development (IRD) Global, Singapore; 4 Department of Epidemiology, O’Donnell School of Public Health, Houston, Texas, USA

## Abstract

**Background:**

Childhood tuberculosis (TB) remains difficult to diagnose, even among exposed household contacts. We assessed age- and sex-specific gaps in TB screening, evaluation, diagnosis, and treatment among child contacts in a high-burden setting.

**Methods:**

As part of a large active screening program in Kotri, Pakistan, from 2014–2016, children aged 0–14 years with household TB exposure were screened for TB disease. We analysed their progression through the TB care cascade and identified factors associated with drop-off at each stage.

**Results:**

Of 3014 child contacts screened, 56.3% were males. Despite clear exposure risk, only 1608 (53.4%) were completely evaluated. Evaluated children were more likely to have lower weight percentiles and report cough, fever, and weight loss (*P* < 0.001). Among those evaluated, 390 (24.3%) were diagnosed with TB (25.8% males *vs.* 22.2% females; *P* = 0.103). Nearly all initiated treatment (98.7%) and completed it successfully (98.2.%) with no significant differences by sex. As age increased, symptom reporting declined into adolescence, and the percentage being evaluated and diagnosed decreased. However, males in certain age groups were significantly more likely to be diagnosed (at age six) or evaluated (at ages seven and 14) than females of the same ages.

**Conclusions:**

The largest gap occurred at the evaluation stage, despite symptoms and known exposure. While treatment initiation and completion were high, observed differences in evaluation completion across age and sex represent a critical barrier to TB elimination in children. Interventions addressing this drop-off – especially among young females – are urgently needed.

Tuberculosis (TB) is an infectious disease caused by the *Mycobacterium tuberculosis* bacterium [1]. Around the world, TB remains in the top-ten killers of children younger than five years [2]. An estimated 7.5 million children are infected with TB each year, and 1.1 million children develop TB disease worldwide, which comprises 12% of the global TB burden [1]. In 2019, an estimated 230 000 children died of TB disease, with over 80% of the deaths occurring among children aged <5 years and 96% among children who did not receive TB treatment [3]. In the past decade, national tuberculosis programs in high-burden TB areas have raised awareness for the challenges of childhood TB [4].

However, diagnosis of TB disease in children remains difficult [3]. Limited capacity of healthcare providers, lack of child health services, shortage of trained clinicians, complex diagnostic algorithms, and limited contact-tracing resources are some factors that make it difficult to accurately diagnose children with TB [5]. Therefore, under-reporting of TB in children is common even when children present to healthcare providers [6]. The World Health Organization (WHO) recommends TB contact tracing as a method for identifying childhood TB [3]. Prior studies emphasise that the diagnosis of any form of TB in any age group should be a qualifier for TB symptom screening and evaluation of all the household members, especially in settings where the lack of health facilities presents systematic barriers for patients to seek care [7]. However, this approach is not widely adopted, especially in high-burden TB countries like Pakistan.

In 2024, Pakistan had a TB incidence rate of 266 per 100 000 [8]. Although contact tracing has been a component of Pakistan’s national TB guidelines since 2007, it has not been systematically implemented [9]. As of 2024, it is estimated that only 5.4% of household contacts of bacteriologically-confirmed TB cases are on preventive treatment [8]. Prior studies in Pakistan revealed that verbal screening and physical evaluation, including a chest x-ray, of children who are household contacts of TB patients was effective in diagnosing children with TB, who were both symptomatic and asymptomatic [9-11]. Therefore, a care cascade comprising six steps (screened, positive screen, evaluated, diagnosed, started treatment, and successful outcomes) was developed to attempt to close the TB diagnosis gap in children in Pakistan [2]. Further research is needed to evaluate the effectiveness of this cascade-of-care framework in detecting and treating children affected by TB among household contacts.

Here, we use data from a large, active screening and contact-tracing project focused on improving childhood TB diagnosis in the Jamshoro district, Pakistan, to describe age- and sex-specific completion of the care cascade among child contacts and quantify differences.

## METHODS

### Study design and participant recruitment

This study was conducted and reported in accordance with the STROBE guidelines for observational studies (Appendix S1 in the **Online Supplementary Document**).

Between 2014 and 2016, we conducted a large-scale active TB screening and contact-tracing project in Jamshoro, a rural district in Pakistan. Children screened for TB included those in contact with adults or children newly diagnosed with TB and seeking treatment at four large public sector hospitals in the district. Adults with TB and the guardians of children diagnosed with TB were asked to bring family members to the health facility for TB screening. Household contacts received two reminder phone calls, one week apart. If the family had not come two weeks after the second reminder, the study team conducted a household visit. Child contacts received chest radiography and blood testing (complete blood count/erythrocyte sedimentation rate). We conducted Xpert MTB/RIF testing (Cepheid, Sunnyvale, California, USA) if children were able to provide a sputum sample. TB diagnosis was made using available clinical parameters, test results, and the clinical judgment of the medical officers evaluating the child. The medical officers at the hospitals were trained in the diagnosis and management of childhood TB by the project’s infectious disease paediatrician and provincial tb program. On-the-job and refresher training were conducted as needed. Medical officers received support with diagnosis and management from the project’s paediatrician through telehealth consultations. For quality assurance, the government district TB coordinator validated individual patient data monthly to ensure that appropriate management guidelines were followed.

A one-time travel reimbursement of PKR 500 (USD 5) was provided to each household that brought family members for screening, and all laboratory testing costs were covered by the project. Further details of the project are provided elsewhere [9].

### Key procedures and measures

We applied a TB cascade framework, consistent with that used in prior paediatric TB studies [10], including six steps for child contacts aged 0–14 years old, spanning from initial screening after their household contact was diagnosed with TB, all the way through to if the child contact was diagnosed with TB themselves and completed treatment.

#### Step one: screening

All children within the data set were household contacts of someone with TB; therefore, all children were screened for TB.

#### Step two: screened positive

As a contact investigation, by definition, was initiated for household contacts of a newly diagnosed TB patient, all children in the household at high risk of TB transmission screened positive and entered the algorithm/framework.

#### Step three: evaluated for TB

We classified children as ‘evaluated for TB’ if they had been seen by a medical officer and had undergone chest x-ray evaluation. Children who had undergone a physical examination, including chest, abdominal, or lymph node examination, were classified as ‘seen by the medical officer.’

#### Step four: diagnosed with TB

Children were classified as ‘diagnosed with TB’ if a medical officer gave a final diagnosis based on either bacteriologic confirmation of TB disease, or a combination of clinical, laboratory, radiological, and/or histopathological test results. Children were classified as having either pulmonary or extrapulmonary TB.

#### Step five: started TB treatment

We referred children who started TB treatment to the national TB control programme and had dates of treatment initiation recorded.

#### Step six: successful TB treatment outcome

We defined a successful TB treatment outcome as either achieving a ‘bacteriologic cure’ or ‘completing TB treatment’ as per the national guidelines.

### Analysis

We calculated the percentage of children who completed each step in the care cascade by dividing the number of children who completed each step by the number of children who completed the prior step. Children were eligible for consideration for each individual step if they reported the outcome/qualified for the prior step. We did this separately by sex (male and female) and age (each year of a child’s age at screening, from zero to 14). We also calculated the percentage of completion for each cascade step for males and females separately, by age group. We used the χ^2^ and Fisher Exact tests to compare the completion frequencies for each cascade step. Additionally, χ^2^ test statistics were obtained to compare the frequency of completion for those who did and did not complete an evaluation by various symptoms, weight percentile, cough, and cough duration. Data were analysed using SAS Studio, v9.4 (SAS, Cary, North Carolina, USA).

## RESULTS

### Aggregate results

During the study period, 3014 child contacts aged 0–14 years were verbally screened for TB disease (**Table 1**). All 3014 (100.0%) child contacts screened met the screening-positive definition, as they were household contacts of newly diagnosed TB patients. A total of 1608 (53.4%) children were then evaluated for TB disease, of which 390 (24.3%) were diagnosed. Of children diagnosed with TB, 385 (98.7%) initiated treatment, and 378 (98.2%) experienced a successful treatment outcome.

**Table 1:** Completed tuberculosis care cascade steps for 3014 child contacts

Step of the cascade completed	n (%)*
Step one: screening	3014
Step two: positive screening	3014 (100.0)
Step three: evaluated for TB	1608 (53.4)
Step four: diagnosed with TB	390 (24.3)
Step five: started TB treatment	385 (98.7)
Step six: successful TB treatment	
*Follow-up lost*	7 (1.8)
*Completed*	378 (98.2)

TB – tuberculosis

*Percentage out of ‘n’ of the previous step.

The mean age of children who were verbally screened for TB was 6.3 years (standard deviation (SD) = 3.8 years). Children aged <5 years made up 36.6% (n = 1103) of all children screened, while children aged five to nine years old accounted for 41.1% (n = 1240), and those aged 10–14 years old accounted for 22.2% (n = 670).

**Figure 1:**
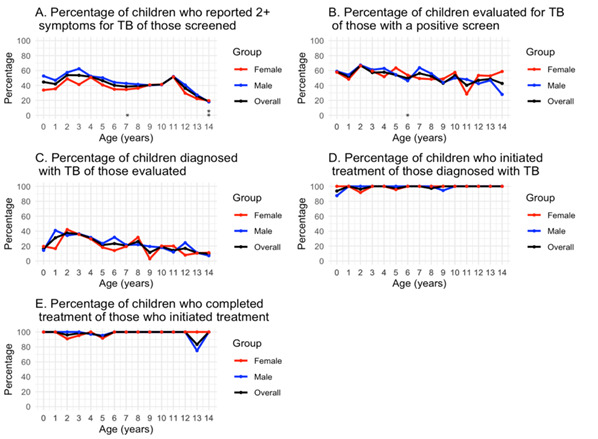
Percentage of children who completed each cascade step by age and gender **Panel A.** Percentage of children who reported two or more symptoms for TB of those who were screened, by age and sex. **Panel B.** Percentage of children evaluated for TB of those with a positive screen, by age and sex. **Panel C.** Percentage of children diagnosed with TB of those evaluated, by age and sex. **Panel D.** Percentage of children who initiated treatment of those diagnosed with TB, by age and sex. **Panel E.** Percentage of children who completed treatment of those who initiated treatment, by age and sex. χ^2^ tests were performed for each age between genders for each cascade step. The black line represents all children in aggregate. The red line represents female children, and the blue line represents male children. *P < 0.05. **P < 0.01. ***P < 0.001. TB – tuberculosis.

Of children evaluated for TB, 922 (57.3%) were male, 416 (25.9%) had a weight-for-age ≤5th percentile, 1166 (72.5%) reported cough, 957 (59.5%) reported fever, and 725 (45.1%) reported weight loss (**Table 2**). Of 3014 children screened (56.3% males), 1302 (43.2% females), (46.7% males *vs.* 38.7% females; *P* < 0.001) reported having two or more TB symptoms (**Figure 1**, Panel A); all were household contacts of individuals diagnosed with TB.

**Table 2:** Characteristics of household contacts by tuberculosis evaluation completion status (n = 3014), n (%)

Variable	Children who completed an evaluation (n = 1608)	Children who did not complete an evaluation (n = 1406)	*P*-value
Male	922 (57.3)	775 (55.1)	
Female	686 (42.7)	631 (44.9)	0.235
Age in years*			
*0*	97 (58.4)	69 (41.6)	
*1*	74 (51.7)	69 (48.3)	
*2*	139 (66.8)	69 (33.2)	
*3*	158 (57.2)	118 (42.8)	
*4*	179 (57.7)	131 (42.3)	
*5*	164 (53.9)	140 (46.1)	
*6*	120 (49.6)	122 (50.4)	
*7*	146 (56.2)	114 (43.8)	
*8*	142 (52.4)	129 (47.6)	
*9*	70 (42.9)	93 (57.1)	
*10*	105 (53.3)	92 (46.7)	
*11*	35 (40.2)	52 (59.8)	
*12*	83 (46.9)	94 (53.1)	
*13*	55 (48.7)	58 (51.3)	
*14*	41 (42.7)	55 (57.3)	<0.001
Weight percentile ≤5	416 (25.9)	207 (14.7)	<0.001
Cough	1166 (72.5)	877 (62.4)	<0.001
Cough duration in weeks			
*<2*	471 (29.3)	567 (40.3)	
*2–3*	311 (19.3)	77 (5.5)	
*>3*	432 (26.9)	233 (16.6)	<0.001
Fever	957 (59.5)	623 (44.3)	<0.001
Weight loss	725 (45.1)	425 (30.2)	<0.001
Presence of BCG scars	779 (48.5)	685 (48.7)	0.887
Pulmonary TB	370 (23.0)	NA	
Extra-pulmonary TB	19 (1.2)	NA	
Family member characteristics†			
*Family history of TB*	940 (58.5)	668 (47.5)	0.034
*TB type in the family is extra-pulmonary*	50 (3.1)	51 (3.6)	0.246
Family member with TB			
*Mother*	462 (28.7)	188 (13.4)	
*Father*	115 (7.2)	144 (10.2)	
*Other*	343 (21.3)	404 (28.7)	<0.001

BCG – bacille Calmette-Guerin vaccine, NA – not available, TB – tuberculosis

*Ages are presented as a percentage of the row totals.

†Includes family members other than the index patient.

### Sex disaggregated results

All of the 1697 males and 1317 females who were verbally screened for TB were categorised as screening positive because they met the criteria of being household contacts of individuals diagnosed with TB. Of these, 922 (54.3%) males and 686 (52.1%) females were evaluated for TB (*P* = 0.235). Among those evaluated, 238 (25.8%) males and 152 (22.2%) females were diagnosed with TB (*P* = 0.103). No differences were observed in treatment initiation or completion by gender (**Table 3**).

**Table 3:** Sex-specific tuberculosis care cascade completion results for 3014 children aged 0–14 y, n (%)

Care cascade step	Overall (n = 3014)	Male (n = 1697)	Female (n = 1317)	*P*-value*
Step one: screening	3014 (100)	1697 (100)	1317 (100)	
Step two: positive screening	3014 (100)	1697 (100)	1317 (100)	
Step three: evaluated for TB	1608 (53.4)	922 (54.3)	686 (52.1)	0.235
Step four: diagnosed with TB	390 (24.3)	238 (25.8)	152 (22.2)	0.103
Step five: started TB treatment	385 (98.7)	235 (98.7)	150 (98.7)	1.00†
Step six: successful TB treatment	378 (98.2)	232 (98.7)	146 (97.3)	0.439†

TB – tuberculosis

*χ^2^ statistical test performed between male and female categories for each cascade step, unless otherwise noted.

†Fisher exact test used.

### Age disaggregated results

Among children evaluated for TB, there was substantial variability in the overall percentage of children aged 0–14 who completed an evaluation, with age 11 the lowest at 40.2% and age two the highest at 68.8% (**Figure 1**, Panel B). Among those aged 14 years, a significantly higher percentage of females (58.7%) completed an evaluation compared to males (28.0%; *P* < 0.01). However, among those aged seven years, a significantly higher percentage of males (63.7%) completed an evaluation compared to females (49.3%; *P* < 0.05) (**Figure 1****,** Panel B). As age increased, there was a general downward trend in the percentage of children diagnosed with TB amongst those evaluated (**Figure 1**, Panel C). A majority of children (n = 238, 61.0%) diagnosed with TB were aged ≤5 years. Among children aged six years, a significantly higher percentage of males (31.8%) were diagnosed with TB compared to their female counterparts (14.0%; *P* < 0.05). The highest percentage of children diagnosed with TB was among females aged two years (42.1%).

As age increased, the percentage of children who initiated treatment remained fairly constant, with the lowest percentage of 93.8% among those aged <1 year (**Figure 1**, Panel D). Among those who started treatment, males and females followed similar trends as age increased, with males having the lowest percentage of 83.3% at age 13. The percentage of children who successfully completed treatment varied from ages one to five, with the lowest overall percentage being at age five (94.1%). For those who successfully completed treatment, males and females across all ages had similar percentages, with the lowest percentage among females aged two years (90.9%) (**Figure 1**, Panel E).

## DISCUSSION

We observed high completion rates for the paediatric TB care cascade among child household contacts, compared with prior literature [2,3,7]. However, our study revealed significant differences by sex and age in the completion of the different cascade steps.

We observed that across several care cascade steps, males comprised a higher percentage of completion compared to their female counterparts. While these differences were not statistically significant, they are potentially operationally important in relation to access or utilisation of services and should be explored further. In developing areas, such as rural Pakistan, barriers limiting access to TB care have sex-related differences that can influence interventions to optimise TB care [12]. It is unclear what is driving the observed sex-differences in this study. However, previous literature has described that these types of differences in TB service completion may be attributed to the following: difficulties accessing TB service sites, stigma surrounding TB diagnosis, TB-related knowledge and education, culture-specific gender roles and beliefs, adherence with national TB program guidelines, and patient satisfaction with TB services [12,13]. Revealing sex-specific gaps is crucial to developing targeted TB interventions that address these drop-offs – especially among young females. Further research should look more closely at drivers of sex-differences among TB care completion for children. Although not statistically significant, there was variability in completion of evaluation, diagnosis, and treatment among adolescents (11–14 years of age) by sex. This may suggest the use of more nuanced care strategies for adolescents, grounded in local context, to support their completion as they progress through the TB care cascade.

Age-related characteristics significantly impact TB screening, evaluation, diagnosis, and treatment outcomes. Young children are most vulnerable to developing severe forms of TB and are over-represented among TB deaths [14]. Despite difficulties in obtaining samples, particularly from younger children, 61% of children diagnosed with TB in our study were aged ≤5 years. The interventions within the paediatric TB care cascade shows promising outcomes for young children.

Consistent with prior studies, our results show that the largest gap in the paediatric TB care cascade occurred at the evaluation stage, despite symptoms and known exposure [10,15]. Some potential explanations for this gap include lack of healthcare access and extreme costs to access health facility including transportation and missed wages for adults accompanying child contacts [15]. Even though the project provided a one-time travel allowance, this may not have offset the total cost incurred. While treatment initiation and completion were high, missed evaluations represent a critical barrier to TB elimination in children.

### Limitations

Limitations of our study include reliance on clinical diagnosis for TB, leading to a possibility of overdiagnosis. To counter this, we built in a robust monitoring and evaluation component, supported by the provincial TB programme. Given the small number of children in individual age groups, we may not have sufficient power to detect differences. Finally, as we only captured the first evaluation in our data as part of the contact investigation, we may have missed children with TB who were diagnosed at appointments presenting outside of contact investigation or who do not engage with the local health system.

## CONCLUSIONS

In this study of the paediatric TB care cascade in Jamshoro district, Pakistan, we identified significant age and sex related differences in the evaluation of child contacts with TB. Interventions that target these age and sex gaps within the TB care cascade framework are necessary to improve the evaluation and treatment outcomes for children. Interventions may need to be tailored to community needs and may include integration with maternal and child health programs, treatment support programs for adolescents (especially boys) and healthcare provider programs to close gaps in paediatric TB care and management. Further research is needed to understand how cultural norms and expectations of gender and age-based roles affect TB care outcomes in Pakistan, specifically.

## Additional material


Online Supplementary Document
